# Stress enhances aggression in male rats with genetic stress hyper‐reactivity

**DOI:** 10.1111/gbb.70005

**Published:** 2024-10-18

**Authors:** Aspen M. Harter, Chris Kim, Anna Yamazaki, Luca Lee, Michelle T. Ji, Mariya Nemesh, Eva E. Redei

**Affiliations:** ^1^ Department of Psychiatry and Behavioral Sciences, Feinberg School of Medicine Northwestern University Chicago Illinois USA

**Keywords:** adolescence, adrenal androgens, aggression, resident‐intruder, restraint stress, testosterone, WMI

## Abstract

The current study investigated stress‐induced aggressive behavior in the resident‐intruder test in males of the genetically stress hyper‐reactive Wistar Kyoto More Immobile (WMI), and the nearly isogenic, control Wistar Kyoto Less Immobile (WLI) strains. Tests were carried out against same‐age intruders during adolescence, and same‐age and juvenile intruders in adulthood. In adolescence and adulthood, prior acute restraint stress decreased social interactions and decreased aggressive behaviors of adolescents and adult WLIs. However, prior stress precipitated aggression in the adult WMI males toward both same‐age, and juvenile intruders compared with control WMIs and WLIs. Trunk blood levels of testosterone and androstenedione increased in stressed WLIs, but not in WMIs, suggesting no direct role of androgens in the increased aggression of WMIs. Expressions of aggression‐relevant genes showed patterns commensurate with being causative in aggressive behavior. The methyl‐CpG binding protein 2 was lower in the frontal cortex of control WMIs, and in the amygdala of stressed WMIs compared with their respective WLIs. Frontal cortex expression of vasopressin receptor 1a and serotonin transporter increased, solely in WMI males after stress. As behaviors were the same toward same‐age and non‐threatening juvenile intruders, the stress‐induced increase in confrontational behavior of the adult WMI male was not because of enhanced fear or anxiety. These results suggest that genetic stress hyper‐reactivity is a risk factor for stress‐induced increases in aggression in males. Additionally, as known aggression‐related genes showed expression patterns paralleling aggressive behavior, this model system could identify novel molecular pathways leading to stress‐enhanced aggression.

## Graphical abstract

Genetically stress hyper‐reactive adult males showed enhanced aggressive behavior, unrelated to fear or anxiety, toward same‐age and nonthreatening juvenile intruders after stress. In contrast, prior stress decreased aggressive behavior of the nearly isogenic control males. Of the aggression related genes, stress‐ and strain‐related changes in the expression of arginine vasopressin receptor 1a and the serotonin transporter are likely causally related to stress‐enhanced aggression in vulnerable individuals.

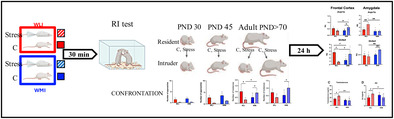

## 1 INTRODUCTION

Exaggerated aggression and violence are common symptoms of many psychiatric disorders, and represent a significant health issue.^1^, ^2^ Development of novel therapeutic strategies would benefit from animal models that can capture components of impulsive or reactive aggression.^3^ Social aggression directed at conspecifics is a highly relevant subtype of aggression in animals and humans.^4^, ^5^ There is a clear sex difference in social aggression, with adult males showing increased social aggression compared with females.^6^ The Challenge Hypothesis by Wingfield and colleagues^7^ proposes that testosterone is the main source of this sex difference in aggressive behavior. Human studies have also shown that increased testosterone is associated with higher levels of aggression.^8^ Adrenal androgens, regulated by the stress responsive hypothalamic–pituitary–adrenal (HPA) axis, have also been associated with aggressive behaviors.^9^, ^10^, ^11^ Thus, stress‐induced adrenal androgens and endogenous testosterone may also be involved in aggressive behavior.

Although aggression is a complex trait, with multiple gene interactions and contributing environmental factors,^12^ studies have suggested specific candidate genes contributing to variation in extreme aggression. A meta‐analysis by Zhang‐James et al.^13^ has shown significant correlations with aggressive tendencies between both humans and animals by monoamine oxidase a (*Maoa*), arginine vasopressin receptor 1A (*Avpr1a*), erb‐b2 receptor tyrosine kinase 4 (*Erbb4*), cell adhesion molecule 1 (*Cadm1*), glutamate ionotropic receptor AMPA type subunit 3 (*Gria3*) and methyl CpG binding protein 2 (*Mecp2*). Another meta‐analysis identified additional genes including the serotonin transporter (*Slc6a4*), cytochrome P450 family 19 subfamily A member 1 (*Cyp19a1*), estrogen receptor 1 (*Esr1*) and estrogen receptor 2 (*Esr2*) with roles in aggression and aggressive tendencies.^14^ Many of these genes show significant activity patterns in the frontal cortex (FCX) and the amygdala (AMY), which are brain regions that play large roles in different forms of aggression.^15^, ^16^, ^17^, ^18^ Larger AMY volume is correlated with increased aggressive responses in humans, and pronounced AMY activity was observed in response to perceived threats, in part through connection to the FCX.^15^, ^16^ The FCX is thought to be the region responsible for the regulation of impulsive behavior, threat response and violent tendencies.^17^ Dysregulation of proper functioning of FCX leads to increases in aggressive behavior.^18^

Human and rodent models show that stress increases aggressive behavior.^24^, ^25^ Previous findings from our lab show that genetic stress hyper‐reactivity can exaggerate the effect of stress on aggression. Adult males of the stress hyper‐reactive Wistar More Immobile (WMI/*Eer*; WMI) strain show aggression toward nonthreatening juvenile intruders after stress, while the control Wistar Less Immobile (WLI/*Eer*; WLI) males do not.^19^ These inbred and nearly isogenic strains^20^ display differences in depression‐like behavior, anxiety and behavioral stress responses.^21^, ^22^, ^23^, ^24^ The differential responses to stress in aggressive behavior toward juveniles in these strains prompted the current study that was aimed to confirm and extend these findings. The previously employed social interaction paradigm^19^ and the resident‐intruder (RI) test provide the same information when the resident animal is in their home cage, and the resident and intruder animals are allowed to interact. Employing the RI paradigm, aggressive behaviors in pre‐, and post‐pubertal resident WLI and WMI males toward juvenile or same‐age intruder conspecifics can be investigated. The study employed the same immediate restraint stress (RS) prior to the RI paradigm as in the previous study.^19^ Peripheral levels of testosterone and adrenal androgens were measured as well as the expression of candidate aggression‐related genes in the amygdala and frontal cortex of adult WLI and WMI males.

We hypothesized that stressed adult WMI males will show increased aggression toward same‐age intruders compared with unstressed WMIs and stressed WLIs, as WMI males already showed stress‐enhanced aggression toward nonthreatening juveniles.^19^ We further hypothesized that stress‐enhanced aggression will only occur in adult but not in adolescent WMI males. The genetic predisposition toward behavioral stress hyper‐reactivity interacting with activation of sex‐, or sex hormone‐specific genes may contribute to the development of aggressive behavior in the WMI males.

## 2 METHODS

### 2.1 Experimental design

**FIGURE 1:**
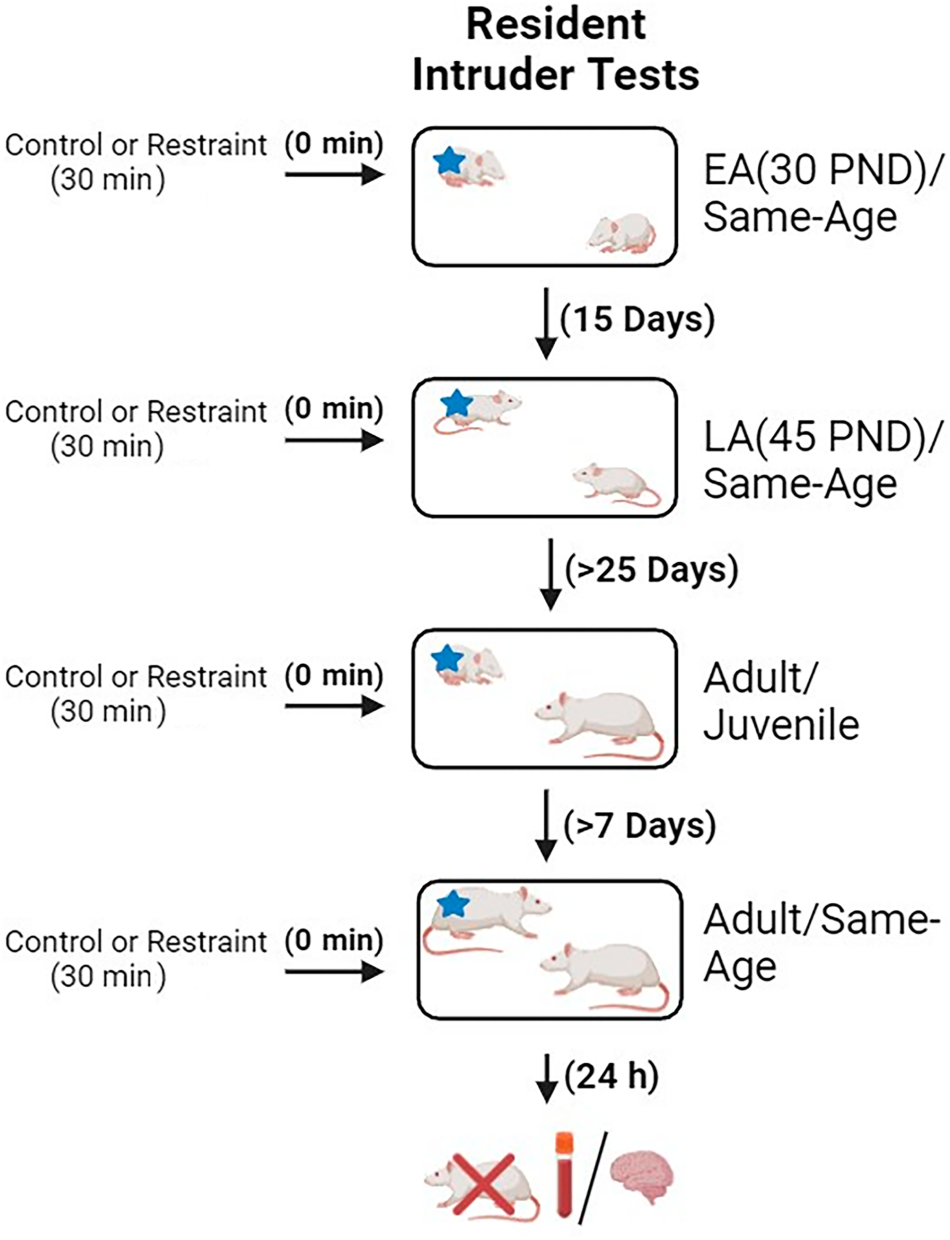
Overview of experimental procedure. Animals were randomly grouped into either a stressed group, that underwent restraint stress for 30 min, or a control group. A resident‐intruder (RI) test was conducted immediately after restraint stress or lack thereof. The RI test was repeated at early adolescence (EA), postnatal day 30 (30 PND), late adolescence (LA; 45 PND), and again >25 days later in adulthood. In adulthood, resident animals were tested first in the presence of juvenile intruders and again at least 1 week later with same‐age adult intruders. At each time, the stressed animals were again restrained for 30 min immediately before the RI test, and controls left undisturbed until the test. Intruder animals are marked with a blue *. Twenty‐four hours after the same‐age adulthood RI test, animals were sacrificed, and whole brains and blood collected.

The experimental design is shown in Figure 1. Animals were single housed 48 h before the experiments started. Half of the animals were exposed to RS immediately before all RI tests, while the other half left undisturbed (nonstressed, control group) consistently throughout the study. The RI tests were conducted at early adolescence (30 days old), and at late adolescence (45–50 days old) with a same‐age conspecific intruder. In adulthood, the RI tests were repeated with juvenile intruders (25–28 days old), and at least 7 days later with a same‐age adult intruder. The nonstressed WLI or WMI intruder animals were assigned to each resident male at early adolescence, based on age and weight match, and were repeatedly used as intruders in adolescence and at the same‐age RI test in adulthood. The rationale to repeatedly use the same intruders were based on the findings that familiar conspecifics lessen the fear responses.^25^ The juveniles used as intruders in the adult RI test were naive unstressed WLI and WMI males. Twenty‐four hours after the adult same‐age RI test, animals were sacrificed via swift decapitation.

### 2.2 Animals

Animals were group housed, 2–3 males per cage depending on the age of the animals, at the Northwestern University Feinberg School of Medicine under the care of the Center for Comparative Medicine. Two days prior to starting the experimental procedures, animals were single housed. Procedures were approved by the Northwestern Institutional Animal Care and Use Committee. Housing was temperature and humidity controlled through a 12‐h light–dark cycle starting at 6:00 AM. Food and water were available ad libitum for all animals. The animals used in the study were inbred male WLI and WMI rats from the 47th–51st generations.

At sacrifice, brains and trunk blood samples were collected. Blood samples were collected into EDTA coated tubes (0.3 μL/0.5 mL whole blood, 0.5 M). The samples were then centrifuged at 4°C and 4000 RPM for 10 min, and the plasma separated for storage at −80°C. Brains were collected in RNAlater™ (Invitrogen, Carlsbad, CA, USA), a solution that stabilizes and protects cellular RNA, and stored at −80°C.

### 2.3 Restraint stress

The RS paradigm was performed in a separate room from the behavioral testing room, as described previously.^19^ Animals were placed into DecapiCone® (Braintree Scientific, Braintree, MA, USA), a plastic tube with openings to allow breathing, and taped down to a surface over the bag to restrain movement. Animals were restrained for 30 min. Immediately following restraint, animals were placed back into their home‐cage and carried to the room where the RI paradigm was conducted. Restraint was only conducted between 10:00 AM and 4:00 PM.

### 2.4 Resident‐intruder test of aggressive behaviors

The same‐age RI tests were conducted in the resident animals' home cage for the stress group immediately after the RS, while the control group underwent the RI tests without prior RS. The intruder rats were males of the same age and weight as the resident animals. To distinguish them in the recordings, their tails were colored with nontoxic markers. The RI test lasted for 10 min, starting when the male conspecific intruder was placed into the cage. Their interactions were recorded. The intruder rat was then removed.

The RI test with juvenile intruder was designed specifically to generate a nonthreatening environment for the resident rat using a much smaller juvenile rat. The juvenile rat thus does not present an anxiety‐provoking threat. The tests were conducted at roughly 75 days of age for the resident rat, at least 7 days before the adult same age RI test. The tests were also conducted in the home cage of the resident animal. The juvenile intruder rat was placed in the home cage of the animal for 4 min. Their interactions were recorded. The intruder rat was then removed.

The tests were scored for specific behaviors of the resident animal. The behaviors scored were grooming, rearing, olfactory investigation (anogenital sniffing) and confrontation, that includes nose‐to‐nose upright threat (fighting), horizontal pushing of the intruder and keeping the intruder down. These measures have been proposed as behaviors displaying aggression in the RI paradigm.^26^ Rearing has been marked as a measure of exploratory behavior, while self‐grooming has been shown to be a measure of stress in rodents.^27^, ^28^ Each behavior was recorded separately and was defined via number of instances. The behaviors were analyzed using multiple counters and repeated viewing. The behaviors were scored by two unbiased researchers with the average score taken for the subsequent analyses. The adult resident animal's behavior toward the same‐age and juvenile intruders have been normalized to behaviors per minute, to compare the same‐age (10 min) versus juvenile (4 min) observations.

### 2.5 Brain dissection and RNA isolation

Collected brains were dissected into coronal slices using a brain matrix (RBM‐4000, Adult Rat Brain Matrix, ASI Instruments, Inc., Warren, MI). Whole frontal cortex and amygdala were then dissected by hand from the relevant slices and collected using the following coordinates as described previously^29^: frontal cortex (AP 5.2–1.7, ML 0–3.3, DV 28 9.0–4.4) and amygdala (AP −0.58 to −2.18, ML 1.5–4.5, DV 4–5.75), and collected into RNAlater™. Tissue was then homogenized using TRI Reagent (Sigma‐Aldrich, Saint Louis, MO, USA) and total RNA was isolated using the Direct‐zol RNA Miniprep Plus kit (Zymo Research, Irvine, CA, USA) according to the manufacturer's protocol. RNA quality and concentration were measured using the Nanodrop 1000 Spectrophotometer (ThermoFisher, Waltham, MA, USA). Samples were considered pure if their 260/280 and 260/230 ratios were in the range of 1.8–2.2. Samples outside of this range underwent a standard RNA precipitation protocol.

### 2.6 Reverse transcription and quantitative polymerase chain reaction

Reverse transcription was carried out on 1 μg of tissue RNA using Superscript VILO™ Master Mix (Invitrogen, Waltham, MA, USA) as directed by the manufacturer. Five nanograms of cDNA/sample/well were analyzed in qPCR using PCR 2X MasterMix Universal for SYBR Green Assay (Lamda Biotech, Saint Louis, MO, USA) in the QuantStudio™ 6 Flex Real‐Time PCR System (Applied Biosystems). Samples were analyzed in triplicates. Primer pairs were designed by Primer‐BLAST (NCBI, Bethesda, MD, USA). Primer sequences are shown in Table S1. Relative quantification (RQ) of transcripts was determined using *Gapdh* as the housekeeping gene and a non‐stressed WLI cDNA sample as the calibrator and calculated by the QuantStudio™ Software in which RQ = 2^−ΔΔCT^.

### 2.7 Plasma hormone assays

Plasma corticosterone (CORT), testosterone, dehydroepiandrosterone (DHEA) and androstenedione (A4) levels were measured by commercially available competitive ELISA kits (Corticosterone Competitive ELISA kit, ThermoFisher, USA; Testosterone ELISA kit, Biomatik, Ontario, Canada; DHEA ELISA kit, Enzo Life Sciences, NY, USA; Androstenedione ELISA kit, ABclonal Technology, MA, USA) according to the manufacturer's protocol. The sensitivities of the assays were: CORT,18.6 pg/mL; testosterone, 49.4 pg/mL; DHEA, 2.9 pg/mL and A4, 78 pg/mL. Plasma samples were diluted to a 1:1000 ratio for CORT, 1:4 for testosterone and DHEA and undiluted for A4. The ELISAs were performed in duplicates. ELISA plates were read on the FLUOstar Omega Microplate Reader (BMG Labtech, Ortenberg, Germany). Standard curves were generated using linear regression on log‐transformed concentration, absorbance data. The resulting equation was then used to calculate the concentration of the hormones in the samples based on absorbance.

### 2.8 Statistics

All statistical analysis was performed using GraphPad Prism version 10.1.3 (GraphPad Software, La Jolla, CA, USA) to determine significant differences between the experimental groups. Significant differences in behaviors were evaluated via a three‐way ANOVA (stress, strain and resident age) for adolescent behaviors and a three‐way ANOVA (stress, strain and intruder age) for adult behaviors. Two‐way ANOVAs (stress and strain) were used to compare hormonal and gene expression data. Post hoc analyses were employed after significant ANOVAs, using the two‐stage linear set‐up procedure of Benjamini et al.^30^ Significance after correction for multiple comparisons was defined as *q* < 0.05, and as *p* < 0.05 for individual *p*‐values. All data was represented as the mean ± standard error of the mean (SEM). ANOVA results are indicated in the results sections, and post hoc analyses are shown in the figures. Linear regression for the analyses of hormone levels were carried out using log transformed concentration of standards. Spearman's correlations were performed to determine associations among behaviors, hormones and genes of interest.

## 3 RESULTS

### 3.1 Resident‐intruder test

**FIGURE 2:**
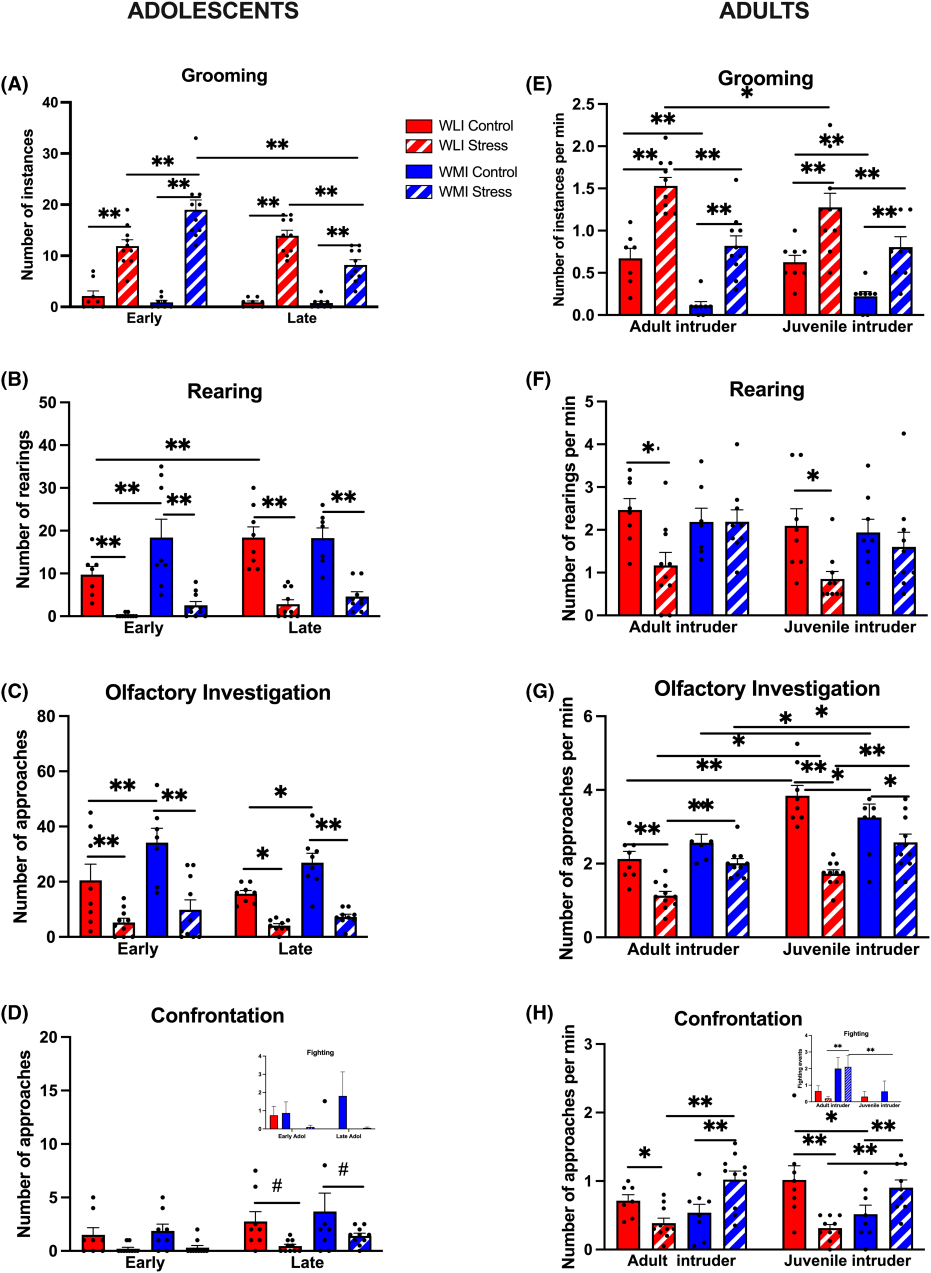
Stress decreased social interaction and confrontation of adolescent males and adult WLIs, but increased aggression of adult WMIs. (A and E) Stress increased self‐grooming in both adolescence and adulthood, regardless of strain. (B and F) Stress decreased rearing in adolescents of both strains, but only in adult WLI males. (C and G) Stress decreased olfactory investigation in both adolescence and in adulthood regardless of strains. (D and H) In adolescence, stress decreased confrontation in both strains. The insert shows no significant differences in fighting between the strains and stress status. In adulthood, stress decreased confrontation in WLIs but increased aggression in WMIs. Adult WMI male fought significantly more with the same‐age intruders after stress compared with stressed WLI males, as shown on the insert. Please note that the overall fighting events were presented, not normalized for the different RI duration with the same‐age or juvenile intruders, because of low fighting events in WLIs. Thus, the difference between adult WMI males fighting with same‐age or juvenile intruders cannot be evaluated. Values shown are as ± SEM; *q < 0.05; **q < 0.01 corrected for multiple comparison, #*p* < 0.05 comparison between specific groups. WLI control *n* = 8; stress *n* = 10; WMI control *n* = 8; stress *n* = 10.

Self‐grooming was used as a measure of perceived stress and anxiety^28^, ^31^ in the resident animals during the RI test. Grooming was observed to be low in both early and late adolescence in control WLI and WMI males (Figure 2A). However, as expected, prior stress exposure increased grooming behavior when compared with controls, regardless of strain, in both early and late adolescence (stress, *F*[1,64] = 223.8, *p* < 0.01). In early adolescence, stressed WMIs groomed significantly more compared with stressed WLIs, while this effect was inverted in late adolescence with stressed WLIs grooming significantly more (age, *F*[1,64] = 9.90, *p* < 0.01; age × stress, *F*[1,64] = 5.27, *p* < 0.05; age × strain, *F*[1,64] = 13.04, *p* < 0.01; age × stress × strain, *F*[1,64] = 18.55, *p* < 0.01).

Grooming during adulthood showed the same pattern as seen in late adolescence, in that stressed WLIs showed increased grooming compared with stressed WMIs, regardless of the intruder's age (strain, *F*[1,62] = 43.31, *p* < 0.01; Figure 2E), and stress increased grooming in both strains, regardless of intruder's age (stress, *F*[1,62] = 73.97, *p* < 0.01).

Adolescent rearing, as a measure of exploratory behavior, showed a somewhat inverted pattern compared with grooming. There was a significantly increased rearing behavior in late adolescence compared with early adolescence (age, *F*[1,61] = 5.48, *p* < 0.05; Figure 2B). In general, adolescent WLIs showed increased rearing activity compared with WMIs, and prior stress significantly decreased rearing (strain, *F*[1,61] = 5.00, *p* < 0.05; stress, *F*[1,61]=93.72, *p* < 0.01).

In contrast, the pronounced stress effect observed in both stages of adolescence was only observed in the rearing of adult WLI males in the presence of either adult or juvenile intruders (Figure 2F). Stressed WLIs but not WMIs showed a significant decrease in rearing compared with their control counterparts regardless of intruder age (stress, *F*[1,62] = 11.06, *p* < 0.01; stress × strain, *F*[1,62] = 6.51, *p* = 0.01).

Anogenital sniffing, termed olfactory investigation, was used as a measure of social interaction based on the sniffing behavior of the resident animal of the intruder. While there was no significant difference in adolescent age, WMI animals showed increased olfactory investigation of intruders compared with WLIs (strain, *F*[1,61] = 13.13, *p* < 0.01; Figure 2C). Social interactions were observed to be significantly decreased following prior stress in both strains when compared with control animals (stress, *F*[1,61] = 61.74, *p* < 0.01).

In adulthood, olfactory investigation was generally increased toward juvenile intruders compared with adult intruders, regardless of strain or stress (intruder age, *F*[1,62] = 34.24, *p* < 0.01; Figure 2G). WLI males investigated the intruders more than WMIs (strain, *F*[1,62] = 6.72, *p* = 0.01). Stress decreased social interaction overall, regardless of strain and the age of the intruder, but this decrease was greater when the resident adults were paired with juvenile intruders (stress, *F*[1,62] = 50.96, *p* < 0.01; stress × strain, *F*[1,62] = 9.56, *p* < 0.01; intruder age × stress, *F*[1,62] = 4.16, *p* < 0.05).

Confrontation as a measure of aggressive behaviors showed no strain differences at any point during adolescence. In general, from early to late adolescence the number of aggressive interactions increased (age, *F*[1,64] = 4.99, *p* < 0.05; Figure 2D), but prior stress significantly decreased confrontation in both strains (stress, *F*[1,64] = 14.26, *p* < 0.01). Measures of fighting was incorporated into confrontation, but it is shown separately as an insert in Figure D. In adolescence, stress decreased fighting regardless of strain or age (stress, *F*[1,64] = 5.78, *p* = 0.01). In adulthood, this stress effect was only observed in WLI males, in contrast, aggressive interactions significantly increased in stressed WMIs compared with WMI controls (stress × strain, *F*[1,62] = 31.28, *p* < 0.01; Figure 2H). As the insert shows, WMI males' instigation of fighting was significantly greater toward the adult intruders compared with juveniles (intruder age, *F*[1,62] = 19.93, *p* < 0.01). WMI males also showed increased fighting compared with WLIs with adult intruders regardless of prior stress, while this pattern was not significant toward the nonthreatening juveniles (strain, *F*[1,62] = 10.03, *p* < 0.01; intruder age × strain, *F*[1,62] = 8.37, *p* < 0.01).

**FIGURE 3:**
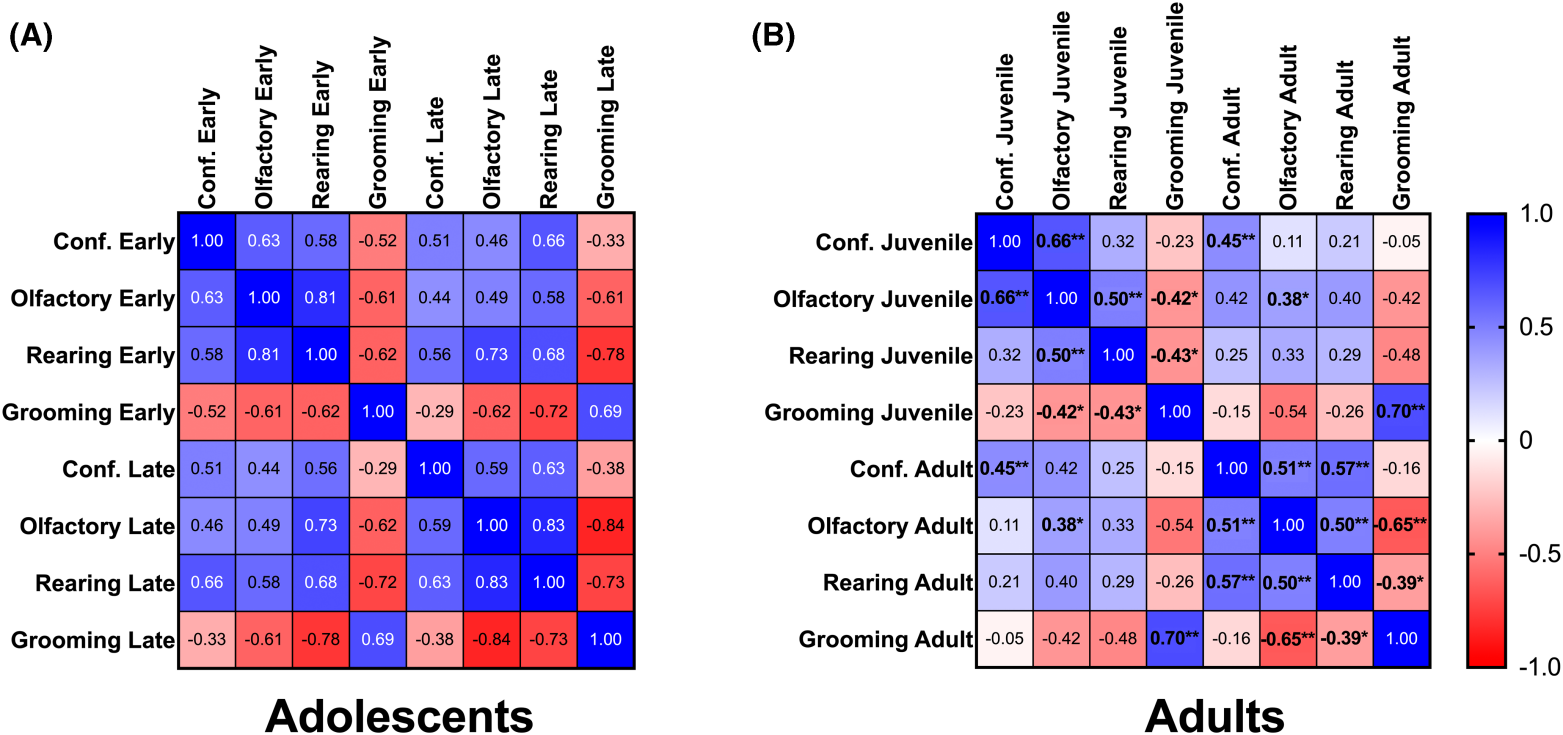
Spearman correlation heatmaps of behaviors during the resident‐intruder test. (A) All behaviors during and between early and late adolescence were significantly correlated with each other except confrontation in late adolescence and grooming in early adolescence. (B) Behaviors in adulthood marked for significance against other behaviors within the same‐age or the juvenile intruder tests. Additionally, significance of the same behaviors between the two tests are noted. An increasing gradient of color is used to express increasing strength of correlations; blue represents positive correlations while red represents negative correlations. Values represent Spearman's *r* values; significance is marked with bolded *r* values and *p < 0.05, **p < 0.01.

Correlations of grooming, rearing, olfactory investigation and confrontation were run separately between early and late adolescents and adults with same‐age and juvenile intruders (Figure 3A,B). Behavioral measures of early and late adolescents toward same‐age intruders correlated significantly with each other (Figure 3A). Grooming was significantly and negatively correlated with confrontation, olfactory investigation and rearing in early, but only with olfactory investigation and rearing in late adolescence. Confrontation was significantly and positively correlated with olfactory investigation and rearing in both early and late adolescents.

Correlations of adult behaviors with same‐aged intruders showed patterns similar to that of late adolescents (Figure 3B). Confrontation, olfactory investigation and rearing were all significantly and positively correlated with each other, while grooming was negatively correlated with olfactory investigation and rearing. This pattern of correlation was mostly maintained in behaviors against juvenile intruders as well, with the exceptions of no significant correlation between confrontation and rearing. Interestingly, behaviors were significantly and positively correlated between adults confronted with same‐age or nonthreatening juvenile intruders, except for rearing.

### 3.2 Plasma corticosterone, dehydroepiandrosterone, testosterone and androstenedione levels

**FIGURE 4:**
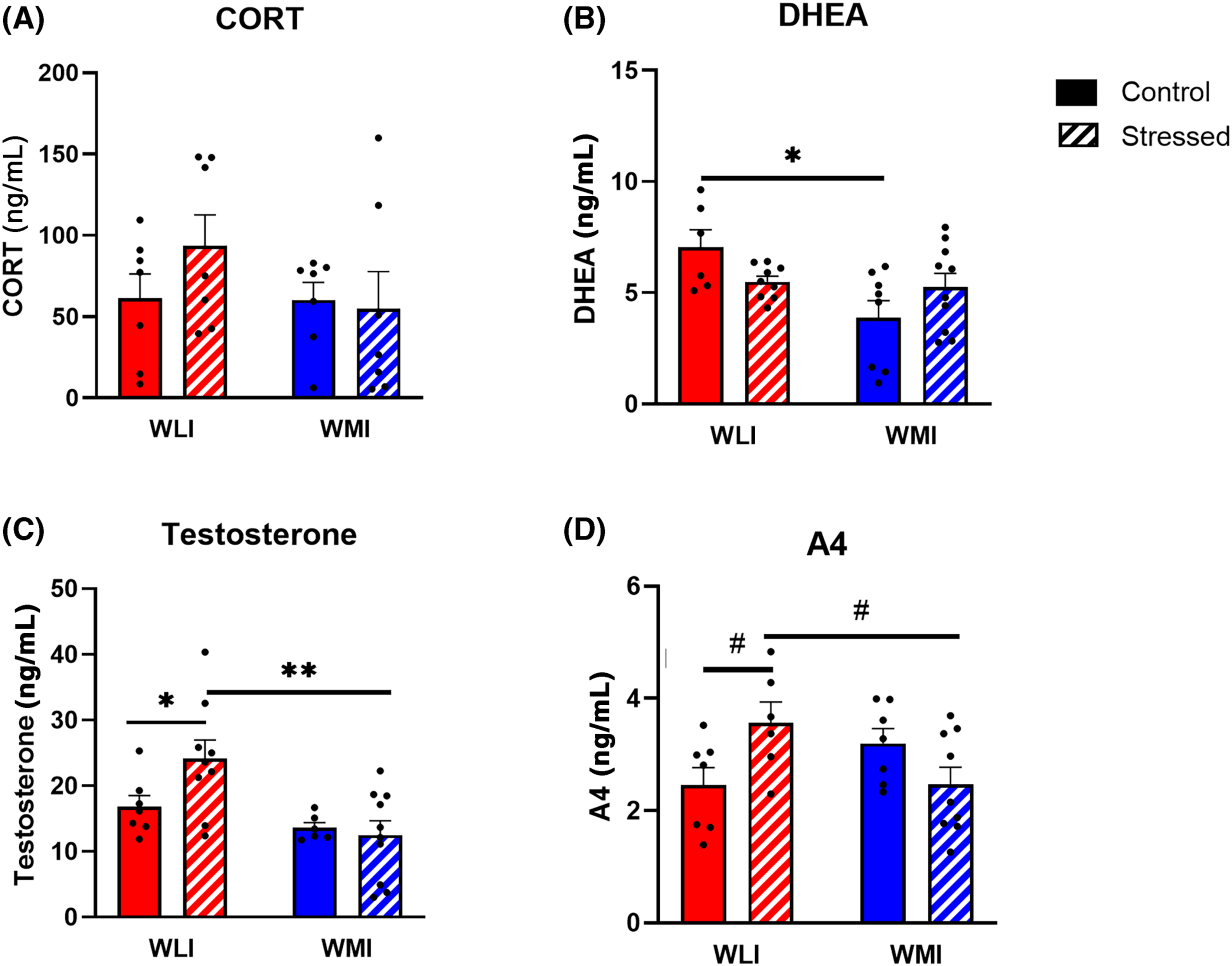
WMI males showed no increases in plasma levels of testosterone or adrenal androgens after the repeated stress of restraint and RI. (A) CORT levels show no difference between strains regardless of stress exposure. (B) DHEA levels were decreased in control WMIs compared with control WLIs. There was no difference between stressed WLI or WMIs. (C) Testosterone levels were higher in WLIs compared with WMI males, and stress increased them further in WLI males, solely. (D) A4 levels increased because of stress in WLI animals, only. Values are mean ± SEM; *q < 0.05; **q < 0.01 after correcting for multiple comparison, #*p* < 0.05 individual group comparisons. Number of animals as in Figure 2.

Plasma CORT levels measured from trunk blood did not differ significantly between or within strains regardless of stress (Figure 4A). In contrast, plasma DHEA levels were significantly higher in control WLIs compared with control WMI males (strain, *F*[1,29] = 7.56, *p* = 0.01; Figure 4B). Stress decreased plasma DHEA in WLIs in contrast to the increase in WMIs compared with their respective controls (strain × stress, *F*[1,29] = 5.57, *p* < 0.05).

Trunk blood testosterone levels were significantly higher in WLIs compared with WMI males (strain, *F*[1,28] = 10.38, *p* < 0.01; Figure 4C). While prior stress increased testosterone levels in WLI males, it had no effect in WMIs. These opposite effects of stress are shown as a trend in the ANOVA analysis (strain × stress, *F*[1,28] = 3.26, *p* = 0.08). Similarly, prior stress increased plasma A4 levels in WLI males while stress tended to decrease it in WMIs compared with their controls (stress × strain, *F*[1,25] = 8.47, *p* < 0.01; Figure 4D).

### 3.3 Gene expression in frontal cortex (FCX) and amygdala (AMY) brain regions

Transcript levels of genes previously associated with aggressive behaviors were measured in brain regions relevant to aggressive behavior: the frontal cortex and amygdala.

#### 3.3.1 Significant differences between groups in both FCX and AMY

**FIGURE 5:**
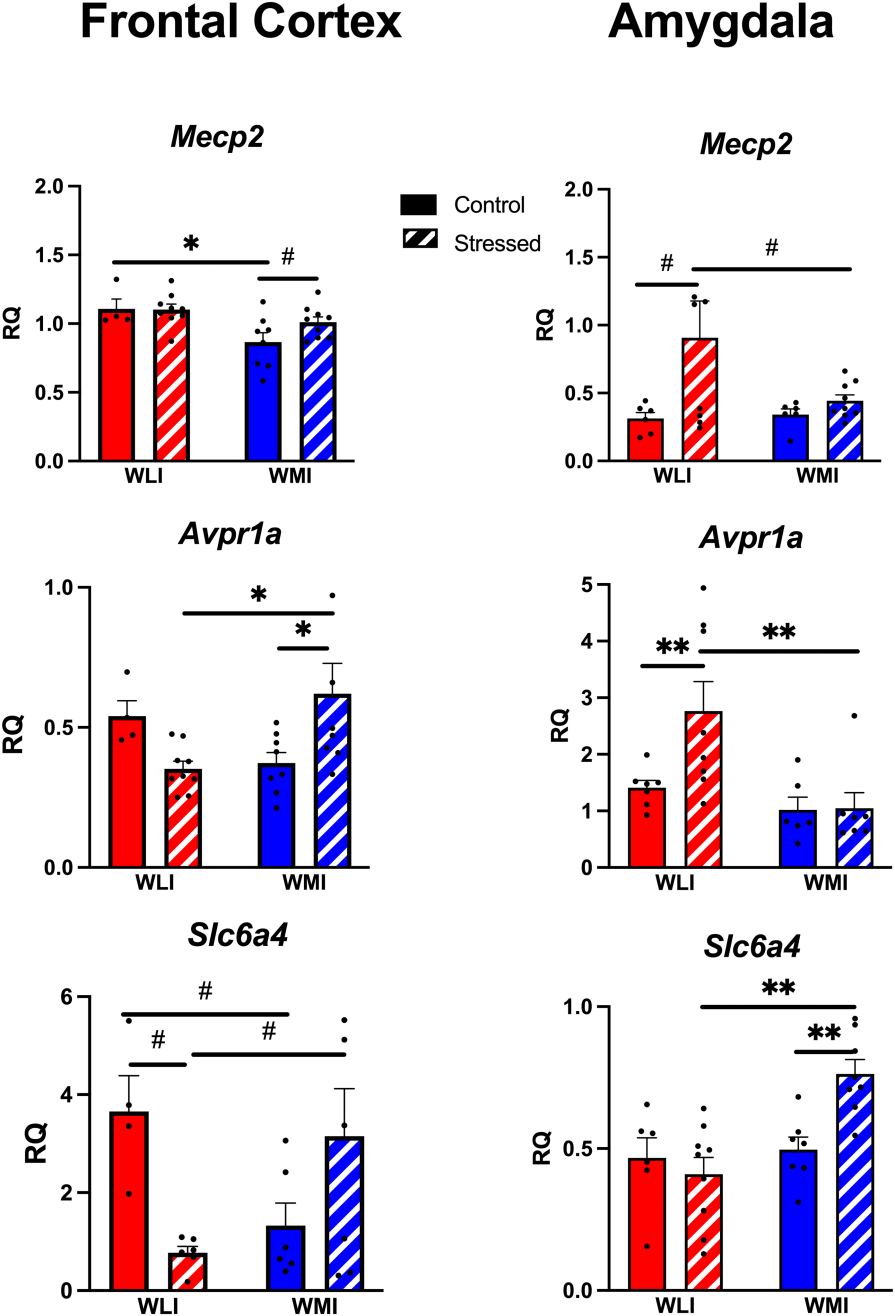
Gene expressions of *Mecp2*, *Avpr1a* and *Slc6a1* were significantly affected by strain and stress, albeit differently in the amygdala (AMY) and the frontal cortex (FCX). In the frontal cortex, *Mecp2, Avpr1a* and *Slc6a4* transcript levels increased after stress, but only in WMIs. In the amygdala, *Mecp2* and *Avpr1a* expressions were increased in stressed WLIs compared with control WLIs and stressed WMI, while amygdalar transcript levels of *Slc6a4* were higher only in stressed WMIs. Values shown are as ±SEM. *q < 0.05; **q < 0.01 corrected for multiple comparison; individual group comparisons #*p* < 0.05, ##*p* < 0.01. Number of animals as in Figure 2.

Gene expression of *Mecp2, Avpr1a* and *Slc6a4* all showed significant differences in expression between groups, both in the FCX and AMY. However, the patterns of transcript level differences varied between the regions (Figure 5). Control WMIs showed decreased expression of *Mecp2* in the FCX compared with the WLI males, with slightly increased levels because of prior stress (strain, *F*[1,26] = 8.92, *p* < 0.01; Figure 5A). In contrast, *Mecp2* expression in the amygdala increased significantly only in stressed WLI males compared with both control WLIs and stressed WMIs (stress, *F*[1,25] = 4.89, *p* < 0.05; Figure 5B). No effect of prior stress was seen in *Mecp2* expression in the amygdala in WMIs.

*Avpr1a* expression was dramatically higher in the AMY than the FCX. *Avpr1a* FCX expression showed opposing results based on strain and stress as transcript levels increased in WMIs because of prior stress but decreased in WLIs. (stress × strain, *F*[1,25] = 9.37, *p* < 0.01; Figure 5C). Interestingly, the pattern of *Avpr1a* expression in the AMY showed similarity to that of *Mecp2* AMY expression; generally higher in WLIs, particularly in stressed WLI males, with no difference by prior stress in WMIs (strain, *F*[1,24] = 8.99, *p* < 0.01; Figure 5D).

Transcript levels of *Slc6a4* in the FCX showed a similar interaction between stress and strain as seen for *Avpr1a* (stress × strain, *F*[1,19] = 10.97, *p* < 0.01; Figure 5E). Transcript levels of *Slc6a4* were lower in control WMIs compared with control WLIs. Inversely, stressed WMIs showed increased FCX *Slc6a4* expression compared with stressed WLIs. This stress‐induced increased expression of *Slc6a4* was also present in the WMI amygdala, with no effect observed in WLIs (strain, *F*[1,26] = 11.13, *p* < 0.01; stress × strain, *F*[1,26] = 8.00, *p* < 0.01; Figure 5F).

#### 3.3.2 Gene expression changes only in FCX or in AMY

**FIGURE 6:**
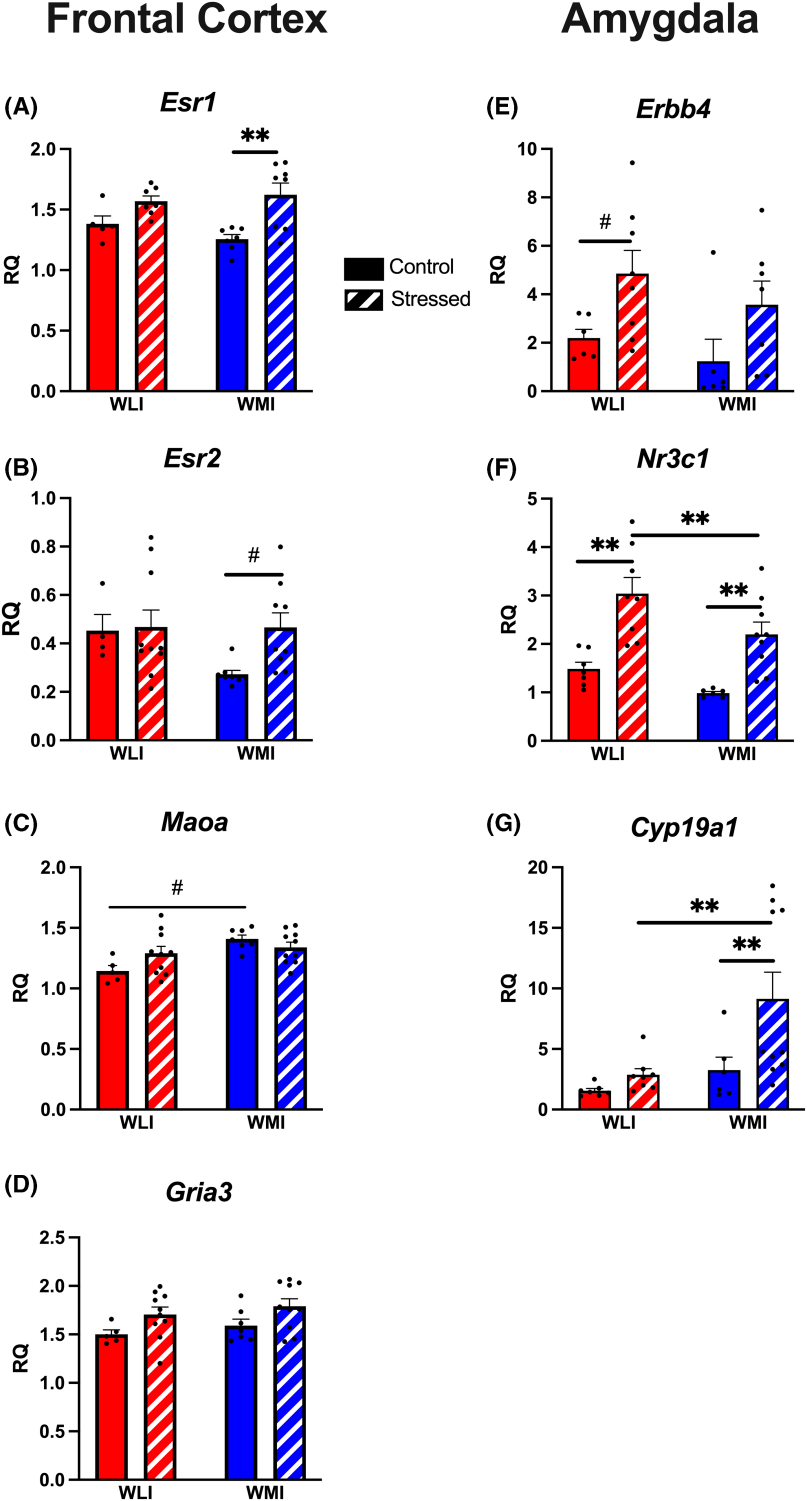
Transcript levels of *Esr1, Esr2, Maoa* and *Gria3* differed by strain or stress only in the frontal cortex, while *Erbb4*, *Nr3c1* and *Cyp19a1* expression differed by strain or stress only in the amygdala. (A and B) *Esr1* and *Esr2* expressions were higher in the stressed WMIs compared with controls. (C) *Maoa* expressions are higher in control WMIs compared with control WLIs. (D) *Gria3* expression levels show the same pattern after stress in both strains. (E and F) Prior stress increased *Erbb4* and *Nr3c1* expressions in both strains. (G) Stress exposure drastically increased *Cyp19a1* expression, but only in the WMIs compared with control WMIs and stressed WLIs. Values shown are as ± SEM. *q < 0.05; **q < 0.01; individual group comparisons #*p* < 0.05, ##*p* < 0.01. Number of animals as in Figure 2.

Gene expressions of *Esr1, Esr2, Maoa* and *Gria3* showed significant effects exclusively in the FCX with no effect observed in the AMY (Figure 6A–D). *Esr1* and *Gria3* expression in the FCX showed a stress effect on both transcripts in a similar fashion (Figure 6A,D). Both genes showed a general increase in expression following stress regardless of strain when compared with controls (*Esr1*, stress, *F*[1,23] = 15.64, *p* < 0.01; *Gria3*, stress, *F*[1,28] = 6.54, *p* = 0.01). Transcript levels of *Esr2* showed so significant differences by strain or stress, although an increase in *Esr2* expression can be observed in stressed WMIs compared with control WMIs (*p* < 0.05). *Maoa* expression was generally higher in WMI males compared with WLIs (strain, *F*[1,28] = 9.27, *p* < 0.01; stress × strain, *F*[1,28] = 4.40, *p* < 0.05; Figure 6C).

Expressions of *Erbb4, Nr3c1* and *Cyp19a1* were observed to show changes between groups exclusively in the AMY (Figure 6E–G). Prior stress increased *Erbb4* expression in the AMY in both strains compared with controls (stress, *F*[1,23] = 7.94, *p* < 0.01; Figure 6E). Expressions of *Nr3c1* and *Cyp19a1* in the amygdala were significantly different between WLIs and WMIs, with the opposite direction, lower *Nr3c1* but higher *Cyp19a1* in WMIs (*Nr3c1*, strain, *F*[1,26] = 7.34, *p* < 0.01; Figure 6F; *Cyp19a1*, strain, *F*[1,27] = 6.65, *p* = 0.01; Figure 6G). This directionality was observed in stress groups as well. Both *Nr3c1* and *Cyp19a1* showed a main effect of stress (*Nr3c1*, stress, *F*[1,26] = 31.19, *p* < 0.01; *Cyp19a1*, stress, *F*[1,27] = 5.42, *p* < 0.05). *Nr3c1* expression was significantly lower, while *Cyp19a1* transcript levels were higher in stressed WMI animals compared with stressed WLIs.

### 3.4 Correlation of aggressive behavior, gene expression and hormones

**FIGURE 7:**
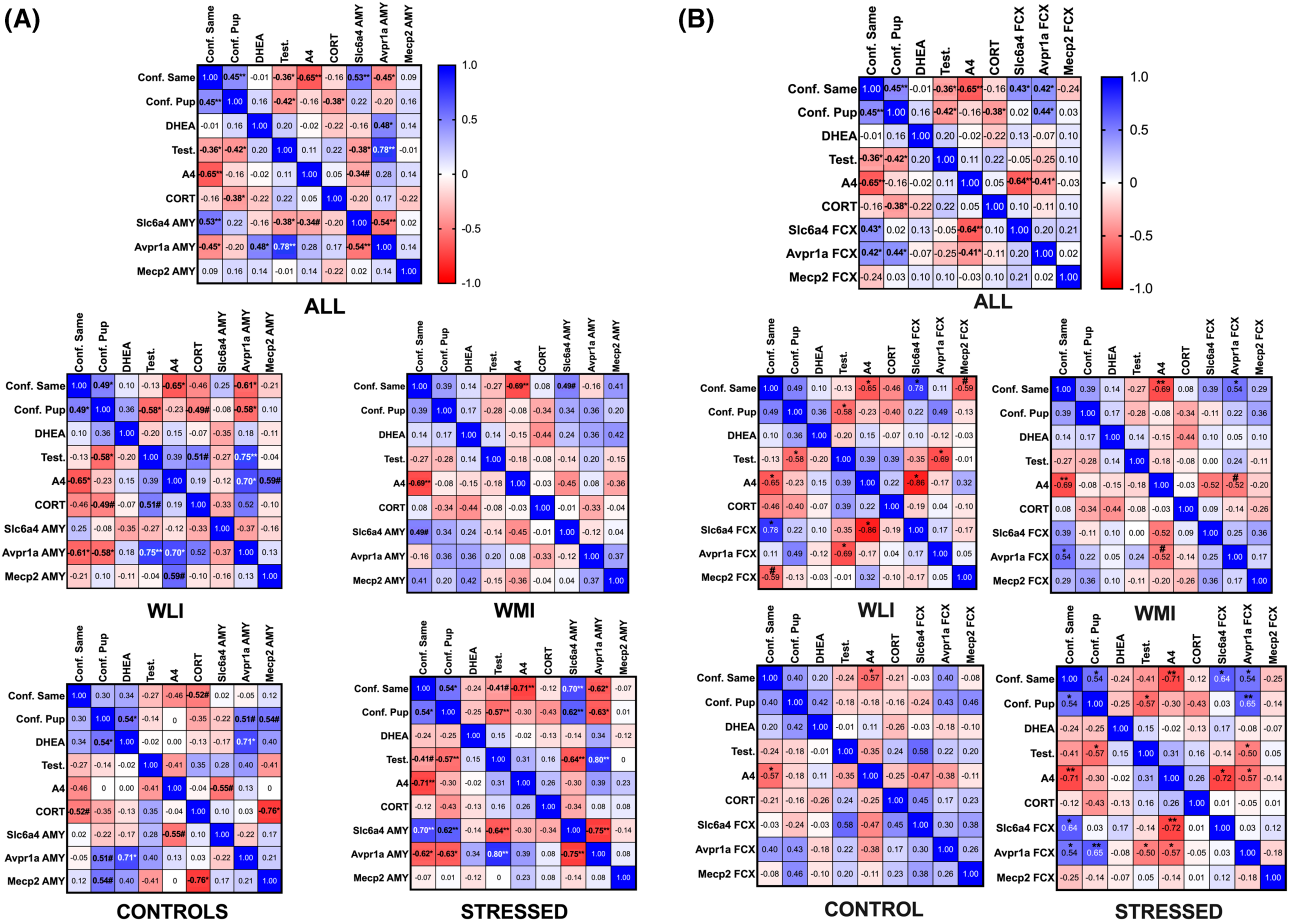
Spearman's correlation heatmaps. (A) Correlations between the behaviors during resident‐intruder test and gene expression in the amygdala and the (B) frontal cortex. An increasing gradient of color is used to express increasing strength of correlations; blue represents positive correlations while red represents negative correlations. Values represent Spearman's *r* values; significance is marked with bolded *r* values and *p < 0.05, **p < 0.01, #*p* < 0.1.

For correlation analyses, we have chosen confrontation with same‐age adult and juvenile intruders, hormone levels and the amygdala and frontal cortex expression of three genes, particularly relevant to aggression (Figure 7A,B). Confrontation with same‐age adult intruders was negatively correlated with plasma A4 levels overall, and in each group regardless of strain and stress status. Confrontation toward the same‐age intruders also correlated negatively with plasma testosterone levels, but only in the overall correlation. However, negative correlations were found between plasma testosterone levels and confrontation toward nonthreatening juveniles, overall, in WLIs, and in the stressed groups as well.

Confrontation correlated significantly, and negatively, with expression of *Avpr1a* in the amygdala; overall with adult intruders, and in the WLI and stressed groups with either age intruders. Inversely, confrontation correlated significantly, and positively, with expression of *Slc6a4* in the amygdala; overall with adult intruders, and in the stressed groups with either age intruders (Figure 7A). In the FCX, confrontation with same age and juvenile intruders correlated positively with *Avpr1a* overall, and in the stressed groups (Figure 7B). The positive overall correlation of confrontation toward same‐age intruders with *Slc6a4* expression in the frontal cortex was significant for the WLI and stressed groups. Thus, *Avpr1a* expression showed brain‐region specific association with confrontation, while *Slc6a4* showed the same positive correlation in both brain regions. It is interesting to note that *Avpr1a* showed greater expression in the amygdala, while *Slc6a4* were more expressed in the FCX (Figure 5).

Significant positive correlation between testosterone and *Avpr1a* gene expression was found in the amygdala, and a negative one in the FCX, while highly significant negative correlation was present between plasma A4 levels and *Slc6a4* expression in the FCX.

## 4 DISCUSSION

The main findings of this study confirm our previous report, namely that genetic stress hyper‐reactivity combined with environmental stressors precipitates aggression in adult males of the WMI strain.^19^ The current study offers that this increased aggressive behavior were not caused by increased anxiety, and did not supplant social behavior of the WMI males. As stress decreased confrontation during adolescence in both strains and in adult WLIs, but increased it for the adult WMIs, testosterone level changes between adolescence and adulthood are not likely the cause of the increased aggression in WMIs. Furthermore, plasma testosterone and adrenal androgen levels were lower in stressed adult WMI males than in WLIs. These results strongly indicate that androgens cannot be directly responsible for the differences in aggression between the strains, or between WMI adolescents and adults after stress. Instead, the results suggest that the enhanced behavioral stress‐reactivity of WMI males affect the expression of brain region‐specific androgen‐regulated or serotonin‐related genes, which contribute to the increased aggression of stressed WMI adults.

Anxiety has been established as a potential cause for increased aggressive behavior in humans.^32^ Social anxiety, among others, is found to be associated with reactive aggression.^33^ Anxiolytic drugs, such as benzodiazepines, have been found to reduce aggressive behaviors,^34^ further suggesting that anxiety may be responsible for the individual variation in aggressive behavior. Findings of the current and previous studies argue against the role of anxiety in the stress‐enhanced aggression of the genetic stress hyper‐reactive WMI male. Adult WMI males show decreased anxiety‐like behaviors compared with WLIs,^35^ and WMI males exposed to stress during adolescence also show decreased anxiety compared with WLIs.^36^ Grooming is thought to be altered by anxiety states and anxiolytic drugs suppress stress‐induced grooming.^27^, ^37^ In the present study, adult control and stressed WMI males showed decreased grooming compared with control and stressed WLIs, respectively. Thus, WMI males seemed to show decreased anxiety similarly to previous observations, and thus the stress‐induced increase of aggressive behavior in adult WMI males is not caused by increased anxiety. Furthermore, the significant correlations between confrontation, olfactory investigation and grooming of adult male residents in the presence of the much smaller juvenile intruders illuminate that fear of the intruder was also not the cause of the increased aggression of the adult WMI male, but likely rather its enhanced stress‐reactivity.

Plasma hormone levels measured at the end of the experiment in trunk blood differed from those in our previous study.^19^ Plasma testosterone and A4 levels were higher in stressed WLIs than in stressed WMIs in the current study, while after the 30 min acute stress testosterone levels were lower in stressed WLIs, but higher in stressed WMI males as shown previously. Both plasma CORT and DHEA increased after stress in the previous study to the same degree in both strains, in contrast to the lack of differences in plasma CORT and the lower levels of DHEA in control WMIs compared with WLIs in the current study. These differences are likely because of the dampening effects of repeated RS and multiple RIs on the CORT and DHEA responses, as it has been shown for repeated stress previously.^38^

Due to the role of androgens in pubertal development and associations with aggression, it is widely accepted as a modulator between pre‐ and post‐pubertal aggressive behavior, particularly in relation to stress.^39^ However, the current study clearly indicates that plasma androgen levels cannot account for the enhanced aggression of adult WMI males after stress. Nevertheless, a percentage of peripheral testosterone and adrenal androgens reaching the brain are aromatized to estradiol, which is known to have major effect on aggressive behavior.^40^ Aromatase, encoded by *Cyp19a1*, control intermale aggression in the amygdala.^41^, ^42^ Testosterone can be either aromatized by *Cyp19a1* to estradiol or 5α‐reduced into the non‐aromatizable dihydrotestosterone (DHT) in target tissues,^43^ but estradiol levels are 10‐fold higher than that of DHT in male rats.^44^ Although A4 is thought of as a weak androgen, it can also be converted by *Cyp19a1* into the weak estrogen, estrone, in the periphery and in the brain, thereby has relevance for aggressive behavior.^45^ The potential role of brain‐derived estrogen in the stress‐induced aggression of WMIs is further supported by the finding of increased FCX expression of both estrogen receptors in WMIs after prior stress in the current study. Whether this is in response to the stress‐induced increase in the expression of the aromatase *Cyp19a1* in the WMI amygdala, or by other mechanism, the greater expressions of *Esr1* and *Esr2* show a parallel to that of enhanced aggression in the stressed WMI males.

Among the other genes known to affect and/or be affected by aggressive behavior, FCX *Mecp2, Avpr1a* and *Slc6a4* showed patterns of strain‐ and stress‐specific expression parallel to aggressive behaviors (Figure 5). In the amygdala, only *Slc6a4* expression paralleled the strain and stress‐specific pattern of aggressive behavior. Impulsive aggression long been attributed to impaired prefrontal inhibition of amygdala function.^17^, ^46^ In contrast, the amygdala is more involved in threat response‐based aggression than FCX.^15^, ^16^ As stress‐induced aggression of the adult WMI males were induced by both potentially threat‐provoking same‐age intruders and by the nonthreat‐provoking juveniles, the WMI aggression is more of a reactive, impulsive response to the intruder.

The differential expression between FCX and AMY in these transcripts could be related to the process of stress‐induced aggression in general. The X‐chromosomal *Mecp2* gene encodes the methyl‐CpG binding protein 2, which can act both as a transcriptional activator and repressor, and thereby influence the expression of many genes.^47^ Expression of *Mecp2* is lower in control WMIs in the FCX, and lower in stressed WMIs in the amygdala compared with their respective WLI groups. *Mecp2* deficiency is known to alter social interaction and enhance aggression.^47^ The effect of variation in *Mecp2* expression is dependent on the genetic background in both animals and humans.^48^

Reinforcing the role of *Avpr1a* in aggression is that its receptor antagonists reduce offensive aggression in male hamsters,^49^ and overexpression of the receptor is associated with higher aggression.^50^ In the current study, expression of *Avpr1a* in the FCX increased solely in the WMI after prior stress. It seems that the FCX *Avpr1a* changes are more in accord with confrontation in the RI test as suggested by the positive correlation between confrontation and *Avpr1a* expression in the frontal cortex. Further suggestion for the importance of FCX comes from a human study that explored the effect of vasopressin (AVP) administration on the frontal cortex‐amygdala circuitry and found no effect on amygdala activation.^51^ The possible interactions between AVP/*Avpr1a* and gonadal hormones in controlling aggression is invoked by the finding that castration, and administration of an androgen receptor antagonist decrease *Avpr1a* expression.^52^, ^53^ The opposite, increased AVP expression is shown after testosterone treatment.^54^ The confirmation of the regulatory role of testosterone on the brain regions specific expression of *Avpr1a* will have to wait for studies directly assess this connection.

The *Slc6a4* gene, as the serotonin transporter, functions as one of the most important biological mechanisms for the availability of serotonin in the synaptic cleft.^55^ Serotonin is believed to be highly relevant to the production of aggression through the “serotonin deficiency hypothesis,” which attributes increased aggression to decreased serotonin levels particularly in the frontal cortex.^56^, ^57^ *Slc6a4* knockout shows decreased aggressive behavior, while increased blood *Slc6a4* expression has been observed in conditions such as antisocial personality disorder.^58^, ^59^ Treatment with selective serotonin reuptake inhibitors reduces impulsive aggression in humans.^60^ The current findings of a positive correlation between *Scl6a4* and aggression agree with the established “serotonin deficiency hypothesis.” The increased expression of *Slc6a4* in both the AMY and the FCX of the stressed WMI would diminish serotonin availability, which might lead to their heightened aggressive confrontations.

There were limitations of this study. The RI test was repeated three times with the same‐age intruders; thus, some familiarity may have developed with the intruders potentially altering behavior toward them. It is suggested by previous findings that familiar conspecifics lessen the fear responses,^25^ but it may not have had major effect in the current study because adult stressed WMIs showed increased confrontation toward the novel juvenile intruders as well, both in the present and previous studies.^19^ The temporal relationship, namely that testosterone levels only increase transiently following the encounter with a conspecific,^61^ suggests that the current study cannot exclude the role of immediate post‐RI rise of testosterone on the aggressiveness in subsequent encounters. Plasma hormone levels are likely not reflecting hormone levels in the relevant brain regions.^44^ Furthermore, as hormone levels and brain regions‐specific gene expressions were measured at the end of the study, association among adolescent RI behavior, hormones and gene expression cannot be ascertained. Additionally, the controls of both strains do not represent genuine experimentally naive animals, as they were exposed to repeated RI tests. Nevertheless, the current results extend previous findings of stress‐induced increases in aggression in the stress hyper‐reactive WMI males, in contrast to the decreased confrontation of the nearly isogenic stressed WLIs. This increased aggression of the WMI male is not related to enhanced anxiety, fear or increased testosterone levels. Instead, aggression‐related and androgen‐regulated genes are likely responsible for the manifestation of aggressive behavior in the stressed and stress‐hyperresponsive WMI males.

## AUTHOR CONTRIBUTIONS

*Conceptualization*: A.H., C.K. and E.E.R. *Methodology and validation*: A.H., C.K., A.Y., L.L., M.J. and M.N. *Analysis*: A.H. and E.E.R. *Writing—original draft preparation*: E.E.R. and A.H. *Writing—review and editing*: A.H., C.K., A.Y., L.L., M.J., M.N. and E.E.R. *Funding acquisition*: E.E.R and A.H. All authors have read and agreed to the published version of the manuscript.

## CONFLICT OF INTEREST STATEMENT

The authors declare no conflict of interest.

## Supporting information


**Table S1.** Primer Sequences for Quantitative PCR.

## Data Availability

The data that support the findings of this study are available on request from the corresponding author.
