# Supplementary material for: Stress enhances aggression in male rats with genetic stress hyper‐reactivity
Source: Genes Brain Behav. 2024 Oct 18;23(5):e70005. doi: 10.1111/gbb.70005 (PMC11487273; doi:10.1111/gbb.70005)
Supplement: Supplementary file 1 — Table S1. Primer Sequences for Quantitative PCR. [file GBB-23-e70005-s001.docx]

**Supplemental Table 1. Primer Sequences for Quantitative PCR**

| **Gene** |  | **Sequence 5’ - 3’** |
| --- | --- | --- |
| *Maoa* | *F* | CCC GAG TCC AAG GAT GTT CC |
|  | *R* | GAT CTT GAG CAG ACC AGG CA |
| *Mecp2* | *F* | ATG TTA GGG CTC AGG GAG GA |
|  | *R* | GTG GGC TGA AGG CTG TAG TG |
| *Esr1* | *F* | GAA AGG CGG GAT ACG AAA AGA |
|  | *R* | TCT GAC GCT TGT GCT TCA ACA |
| *Esr2* | *F* | CAT CAG TAA CAA GGG CAT GGA A |
|  | *R* | CAC CGG GAC CAC ATT TTT G |
| *Nr3c1* | *F* | AAC AGA CTT TCG GCT TCT GGA A |
|  | *R* | TGG AAC GCT GGT CGA CCT AT |
| *Gria3* | *F* | CTC CGG GAG TAA GGA CAA GA |
|  | *R* | TGG ACT CTG CCC GTG ATT TG |
| *Erbb4* | *F* | CCC CCA TGT CGG GAA ATC AG |
|  | *R* | GGC ATG GGC ATT CCT TGT TG |
| *Cadm1* | *F* | TCC TGG TCC CTC CAC GTA AC |
|  | *R* | TCA ATC TCC CCT TCA ACT G |
| *Avpr1a* | *F* | AAG CGC CTA CAT CCT TTG CT |
|  | *R* | TGG AAG GGT TTT CTG AAT CGG T |
| *Slc6a4* | *F* | TGA GGA GTT CTA CTT GCG CC |
|  | *R* | ACA CCC CTG TCT CCA AGA GT |
| *Cyp19a1* | *F* | GAT TTT CGC TGA GAG ACG TGG |
|  | *R* | GTG ACG GAC ATG GTG TCA GG |
| *Gapdh* | *F* | CAA CTC CCT CAA GAT TGT CAG CAA |
|  | *R* | GGC ATG GAC TGT GGT CAT GA |
